# Primary linitis plastica of the entire colon in a background of ulcerative colitis: a case report

**DOI:** 10.1186/s12957-014-0425-1

**Published:** 2015-02-04

**Authors:** Adina E Feinberg, Maja Barnard, Stanley M Feinberg

**Affiliations:** Department of Surgery, University of Toronto, Toronto, ON Canada; Department of Laboratory Medicine and Pathobiology, University of Toronto, Toronto, ON Canada; Division of General Surgery, North York General Hospital, Toronto, ON M2K 1E1 Canada; Department of Laboratory Medicine, North York General Hospital, Toronto, ON M2K 1E1 Canada

**Keywords:** Linitis plastica, Colorectal cancer, Ulcerative colitis, Signet ring cell

## Abstract

We present a case report of colorectal cancer arising in a young patient with ulcerative colitis of only 6 years duration. The pathology was unusual with extensive pancolonic involvement in a lintitis plastica fashion. This case represents a clinical example where colon cancer occurred prior to the onset of recommended screening according to guidelines regarding patients with ulcerative colitis.

## Background

Colorectal cancer is a serious outcome associated with ulcerative colitis. However, this risk is correlated with the duration of inflammation. Current data estimate that the risk of colorectal cancer in ulcerative colitis is 2% at 10 years, 8% at 20 years, and 18% at 30 years [1]. Signet ring cell carcinoma is an uncommon form of cancer in the colon and rectum, representing 1% of cancers in one series [2]. We present a case of poorly differentiated colonic adenocarcinoma with focal signet ring cell features involving virtually the entire colon in a linitis plastica fashion, which may be only the second such case recorded in the literature [3]. Additionally, our patient was a 20-year-old female with only a 6-year history of ulcerative colitis.

## Case presentation

**Figure 1:**
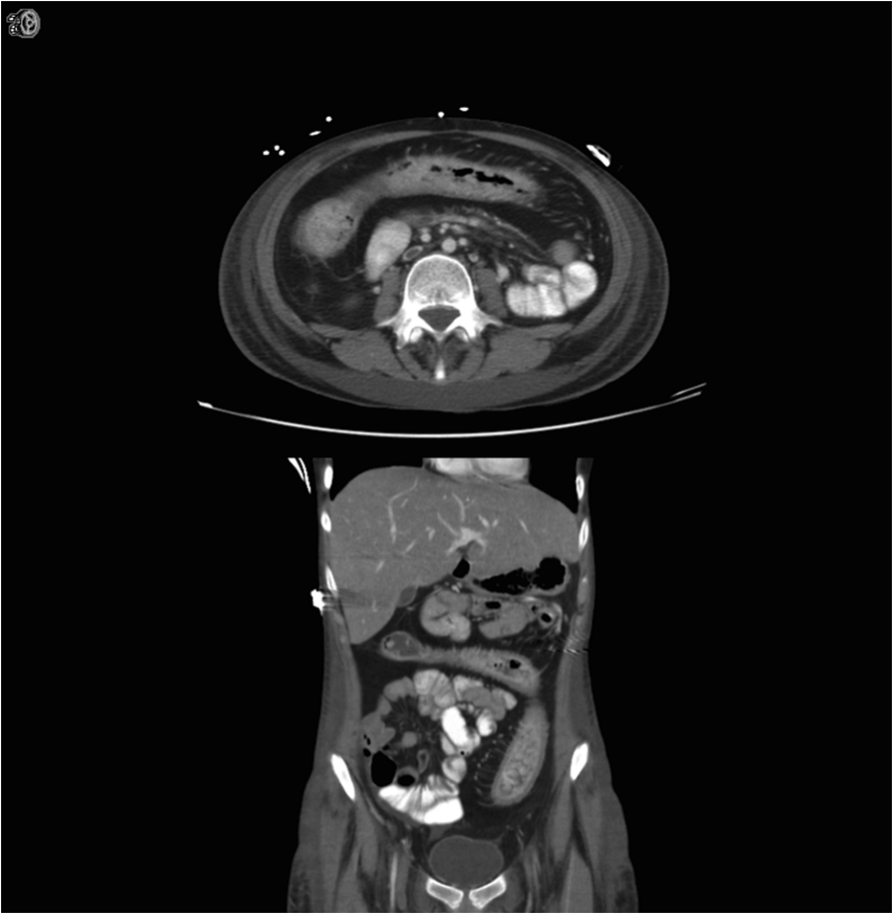
**Computed tomography scan demonstrating diffuse colonic thickening.**

The patient presented in May 2013, with dehydration occurring in the context of a 6-year history of poorly controlled ulcerative colitis. The patient was very resistant to the recommendation of surgery. Despite trials of steroids, 5-aminosalicylic acid, and anti-tumor necrosis factor agents, she continued to have bowel movements up to ten times per day. She had a several-year history of amenorrhea. Her weight was 38 kg. She had anasarca, a serum sodium of 121 mmol/L, a creatinine level of 34 μmol/L, and an albumin level of 10 g/L. Her hemoglobin was 94 g/L, and her white blood cell count was 7.6 × 10^9^/L. Imaging revealed pancolitis (Figure 1). On endoscopy, her colon was markedly foreshortened and straight measuring 45 cm to the ileocecal valve. The entire colon was filled with pseudopolyps, with no obvious malignancy. Many biopsies were taken throughout the colon and rectum. All biopsies were positive for poorly differentiated adenocarcinoma with focal signet ring cell differentiation. Her carcinoembryonic antigen level was within normal range at 1.1 μg/L. There was no sign of distant metastasis on radiologic staging. She was optimized preoperatively with 10 days of parenteral nutritional support. Subsequently, the patient proceeded to surgery. Since biopsies low down in the rectum had been positive for rectal cancer, there were no plans to perform a sphincter-sparing procedure. Traditionally, when operating on a debilitated patient with colitis, the initial operation is a subtotal colectomy. In this patient, due to the pancolonic nature of the cancer, the decision was made to perform a total proctocolectomy to avoid transecting malignancy. Initially, the surgeon began with a laparoscopic approach but conversion to an open procedure occurred early, as the bowel was markedly thickened and boggy, causing concern that there could be potential spillage. The patient’s postoperative course was unremarkable.

**Figure 2:**
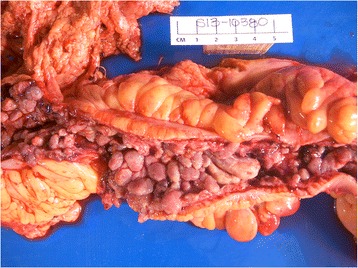
**Gross appearance of the colon.**

**Figure 3:**
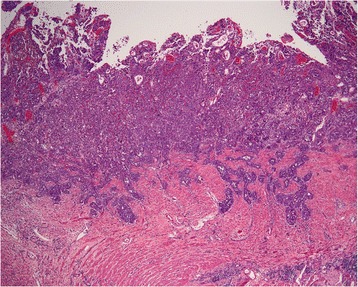
**Microscopic image of poorly differentiated adenocarcinoma invading into submucosa.**

Pathologic findings confirmed that there was continuous involvement of the mucosa of the entire colon with flattening and multiple pseudopolyps (Figure 2). No discrete tumor was identified. On microscopic examination, there was poorly differentiated invasive adenocarcinoma with focal signet ring cell features from the cecum to the dentate line. This also extended into the ileocecal valve and distal terminal ileum. This diffuse infiltration pattern is known as linitis plastica. The resection margins were all negative. It was very unusual that virtually the entire mucosal surface was involved with cancer. There was invasion into the submucosa, classifying the lesion as pT1 (Figure 3). Small intervening areas of uninvolved mucosa showed histological features of chronic colitis with foci of flat and polypoid high-grade dysplasia. Eighty-two lymph nodes were retrieved with the specimen and sixty-five of them contained metastatic adenocarcinoma. The pathologic findings were confirmed separately at the University Health Network in Toronto, Canada.

The patient completed twelve cycles of adjuvant FOLFOX over 6 months. She is currently disease free.

## Conclusion

Colonic linitis plastica is extremely rare, estimated at less than 0.1% of colon cancers [4]. Secondary linitis plastica of the colon is more common and the treating team should consider investigating for a primary tumor.

It is difficult to determine when to initiate screening colonoscopy for colorectal cancer detection in patients with ulcerative colitis. Prior reports of low incidence of malignancy among patients without longstanding colitis [1] lend to establishing guidelines that use duration of disease as a criterion. This is problematic since there is a distinction between the initiation of symptoms and the official diagnosis. Nonetheless, major American and international associations have published guidelines that recommend screening for dysplasia should begin after 8 years of disease activity in ulcerative colitis [5-7]. There are specific stipulations for patients with pancolitis or primary sclerosing cholangitis, but none of the guidelines recommend initiating screening prior to the 8-year mark. This case challenges those guidelines, as the patient presented with extensive colonic neoplasia after just 6 years of disease activity.

Despite a low overall risk of colorectal cancer in patients with ulcerative colitis of relatively short duration, there is evidence that malignancies may be missed in that initial window. A retrospective Dutch nationwide population-based analysis found that approximately 20% of colorectal cancer cases in patients with ulcerative colitis presented prior to the recommended initiation of screening colonoscopy [8]. Thus, it may not be adequate to use duration of disease as an isolated clinical marker for screening guidelines. Other factors such as severity of disease [9] and presence of pseudopolyps [10] contribute to increase the risk of developing colorectal cancer. It is also possible that the immunosuppressive effect of long term anti-tumor necrosis factor therapy may have been a predisposing factor. We propose initiating screening for colorectal cancer in ulcerative colitis earlier in patients that have these worrisome features. In those without reasonable symptom control, colonoscopy should be performed regularly. Furthermore, altering the medical treatment or surgical intervention should be considered. In our patient, optimal care would have entailed subtotal colectomy prior to diagnosing malignancy simply on the basis of refractory disease with severe malnutrition.

## Consent

Written informed consent was obtained from the patient for publication of this Case report and any accompanying images. A copy of the written consent is available for review by the Editor-in-Chief of this journal.
